# The WHO South-East Asia Region Nutrient Profile Model Is Quite Appropriate for India: An Exploration of 31,516 Food Products

**DOI:** 10.3390/nu13082799

**Published:** 2021-08-15

**Authors:** Chandra Pandav, Lindsey Smith Taillie, Donna R. Miles, Bridget A. Hollingsworth, Barry M. Popkin

**Affiliations:** 1 Centre for Community Medicine, All India Institute of Medical Sciences (AIIMS), New Delhi 110029, India; pandavcs@hotmail.com; 2 Department of Nutrition and Carolina Population Center, The University of North Carolina at Chapel Hill, CB #8120, 123 West Franklin Street, Chapel Hill, NC 27516, USA; taillie@unc.edu; 3 Carolina Population Center, The University of North Carolina at Chapel Hill, CB #8120, 123 West Franklin Street, Chapel Hill, NC 27516, USA; drmiles@email.unc.edu (D.R.M.); bhollin@email.unc.edu (B.A.H.)

**Keywords:** nutrient profile models, nutrient profiling, front-of-package labels, warning labels, ultra-processed foods, noncommunicable diseases, India

## Abstract

The rapid rise in prevalence of overweight/obesity, as well as high prevalence of type 2 diabetes and other nutrition-related noncommunicable diseases, has led the Food Safety and Standards Authority of India (FSSAI) to propose a front-of-package labeling (FOPL) regulation. An effective FOPL system applies a nutrient profile model that identifies foods high in sugar, sodium, and saturated fat that would receive a warning label for consumers to effectively discern between more and less healthy foods. Previous Nutrition Alchemy data collected by the food industry (*n* = 1306 products) estimated that approximately 96% of foods in India would have at least one warning label based on the FSSAI proposed FOPL. This near universal coverage of warning labels may be inaccurate and misleading. To address this, the current study compared two nutrient profile models, the WHO South-East Asia Region Organization (SEARO) and the Chilean Warning Octagon (CWO) Phase 3, applied to food products available in the Indian market from 2015–2020, collected through Mintel Global New Products Database (*n* = 10,501 products). Results suggest that 68% of foods and beverages would have at least one ‘ high-in’ level warning label. This study highlights the need to include a more comprehensive sample of food products for assessing the value of warning labels.

## 1. Introduction

India faces a major epidemic of diabetes and overweight/obesity among adults, and a growing child obesity problem coupled with a significant problem of stunting and undernutrition [1,2]. Related is the ever-increasing burden of diabetes and other nutrition-related noncommunicable diseases [3,4,5]. At the same time, the packaged and processed food supply in India is ranked as one of the worst in the world [6]. High intakes of unhealthy packaged and processed foods are major drivers of overweight and obesity for both children and adults [7,8]. As revealed in a speech made by Prime Minister Modi, the Indian government has aspirations for promoting globally the country’s success in preventive healthcare [9]. To achieve this goal, there is only one major option available as regards interventions proven to promote changes in the packaged processed food supply via reformulation as well as obtaining major changes in purchasing by all socioeconomic classes [10]. Front-of-package labeling (FOPL) systems have been implemented in many countries and are proven to decrease purchases of foods with unhealthy levels of sugar, sodium, and saturated fat.

**Figure 1:**
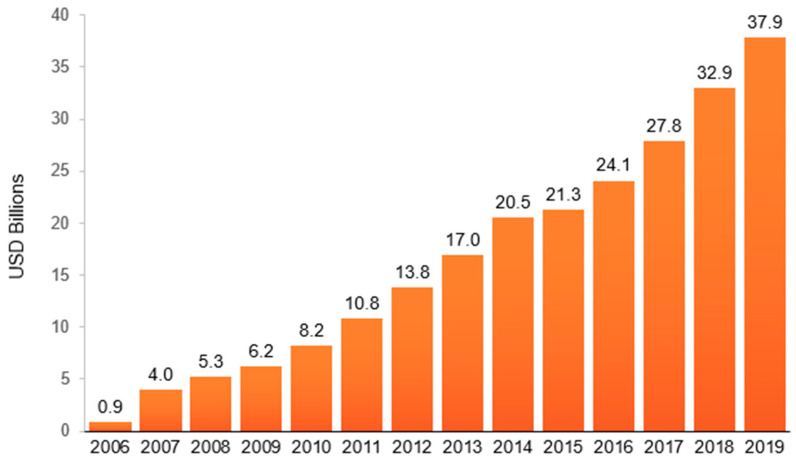
Retail value* of packaged junk foods and soft drinks** in India from 2006–2019 (In USD billions; Rupee and Dollar values in terms of Indian Consumer price index 2006 = 1.00). *Retail selling price, i.e., sales at end-price to consumers, including retailer and wholesaler mark-ups and sales tax (except in the US and Canada) and excise taxes. **Junk food and soft drink categories include: cakes, pastries, confectionery (chocolate, sugar confectioneries, and gum), savory snacks (nuts, seeds, trail mixes, salty snacks, savory biscuits, popcorn, pretzels, and other savory snacks), instant noodles, sweet snacks (fruit snacks, snack bars, sweet biscuits, and ice cream), carbonates, concentrates, juice drinks, and nectars (excluding 100% juices), ready-to-drink coffees and teas, energy drinks, and sports drinks. Data source; Euromonitor International Limited 2021 © All rights reserved.

India faces one of the most rapidly growing food supplies in unhealthy ultra-processed junk food and beverages (i.e., items high in added sugar, added sodium, or added saturated fats) in the world [11,12]. Euromonitor sales figures from 2006–2019, presented in Figure 1, highlight the growth of such foods in India [13]. The retail value of packaged junk foods and soft drinks in India grew by 42.1 times in just 13 years, from US $0.9 billion in 2006 to over US $37.9 billion in 2019 (in 2006 rupees are used to show comparable growth), and continues to grow rapidly [14].

It is important to note these same ultra-processed unhealthy foods are also fed to infants and preschoolers [12]. In Nepal one study showed among poor rural preschoolers, 25% of their calories came from these junk foods [13]. Across the globe, malnourished infants and preschoolers are being fed these ultra-processed foods at an ever-increasing rate. [12]. Thus, these ultra-processed foods impact both the growing obesity and diabetes crises in India, and the stunting and hunger of children through the provision of empty calories (calories coming from foods with little to no nutritional value) [8,15,16,17,18].

The Food Safety and Standards Authority of India (FSSAI) has proposed that India institute a warning system of front-of-package labelling (FOPL). This is extremely wise and laudable, as research on actual purchases and reformulation show that a mandatory warning system is the most effective approach for changing food reformulation and purchasing at the national level. Voluntary systems, including the UK’s traffic light system and Australia’s healthy stars, have not shown any impact on food purchasing and a very small impact on reformulation. In contrast, Chile was the first country to introduce a front-of-package warning label system comprising of black octagonal labels covering 10% of the main face of the label on all packaged products [19]. Chilean Warning Octagons (CWO) identify products high in added sugar, added sodium (i.e., salt based on sodium), and added saturated fats, as well as including a label for high calorie products with these added nutrients. These warning labels were also used to ban labeled food from advertising to children via all media, and from selling or offering in schools [19]. After the first phase of the CWO law, there was a 24% decrease in purchases of sugar-sweetened beverages and a significant but smaller decreased in total calories (49 kcal/capita/day), sugar (20.7 kcal/cap/day), and sodium (96.6 mg/cap/day) consumed from unhealthy ‘high-in’ foods and beverages with warning labels [10,19,20]. The CWO also helped children and their parents better identify unhealthy foods and discourage them from consuming them [20]. Thus, as the World Bank notes and recommends, these policies are mutually reinforcing [21].

One key to Chile’s success was the use of a strong nutrient profile model (NPM). NPMs are systematic approaches to classifying foods based on their nutrient content, ingredients, or other characteristics (e.g., processing levels or basic vs. discretionary foods). For front-of-package labeling systems, the underlying NPM is important because it dictates which products receive a label within the warning label system, and accurately categorizes products that have high levels of nutrients of concern, such as sugar, sodium, or saturated fat. On the other hand, the percentage of products covered by an NPM is also important, since if too few or too many products are covered, it will be difficult for consumers to use the labels to distinguish between more vs. less unhealthy products. The CWO system was built on a strong NPM comparable to the WHO model for South-East Asia, though with one set of thresholds for all foods and a separate set of thresholds for all beverages [22]. Researchers investigated the distribution of nutrients in the food supply to determine thresholds for each nutrient of concern [19]. Prior to the implementation of the law, approximately 51% of Chile’s food supply exceeded thresholds and was eligible to receive a warning label octagon [23].

The WHO South-East Asia Region Organization (SEARO) have developed an NPM that focuses on foods high in added sugar, sodium, saturated fats, total fats and total sugar, and energy density [24]. This NPM was created as a basis for foods which should be banned from advertisement to children based on their unhealthy food content. The WHO SEARO used standardized food groupings used by other WHO regional offices and has tested nutrient cutoffs among four other countries within the region. This study was pretested on samples of data from five countries, including India, Indonesia, the Maldives, Myanmar, and Sri Lanka. Subsequently, India-based food companies provided selected nutrition facts panel data from foods to the Nutrition Alchemy, a company in Mumbai that consulted for the FSSAI to test the SEARO model [23]. They utilized a small database of industry-selected products that numbered 1306. One concern with this approach is that if the sample is not large enough, it will not represent the underlying food supply and not accurately estimate what proportion of the food supply will meet criteria under different NPMs.

In this short paper, a database of 31,519 foods and beverages from the Mintel Global New Products Database was reviewed. Because India lacks mandatory reporting of nutrients on the nutrition facts panel, many products were missing one or more key ingredient, thus, a sample of 10,501 products included enough information for submission to NPM testing. This provided us with a set of all packaged processed foods in India’s food supply with data available, in contrast to the small sample of products from the self-selected industry data. What follows are a brief methods section, the results, and a discussion of their implications for India to follow either the SEARO model or CWO.

Our objective in this paper is to use a large database of products with full nutrient measures covering a range of food categories and apply the SEARO NPM to determine foods which would meet criteria for a FOP warning label. Results of the current sample will then be compared with the Nutrition Alchemy study. In addition, we examine results applying two NPMs: the SEARO and the Chilean Warning Octagon Phase 3.

## 2. Materials and Methods

### 2.1. The Data

**Figure 2:**
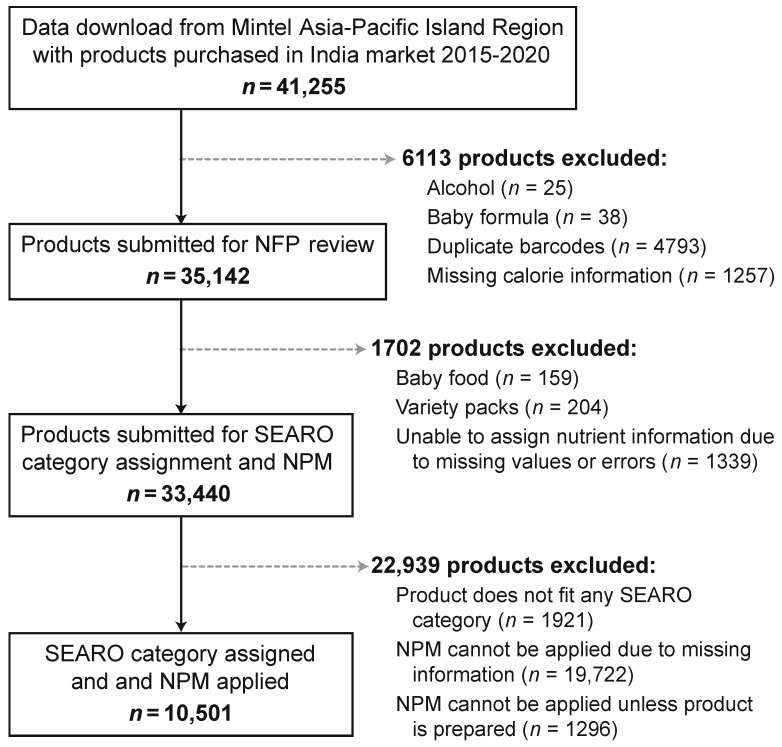
Flow diagram representing the initial and final dataset and reasons for exclusion. Note: NFP = nutrition facts panel; SEARO = South-East Asia Region Organization; NPM = nutrient profile model.

Food product data from 2015–2020 was downloaded from the Mintel Global New Products Database (GNPD) Asia-Pacific Island Region, which collects nutrition information on food and beverages available in the Indian market, including products that are new, reformulated, or have had any packaging changes (e.g., a shift in colors, promotional strategies, or other on-pack marketing elements) [25]. Figure 2 provides a flow chart detailing the process of creating the data set for analysis. The raw data downloaded from Mintel GNPD included 41,255 food and beverages, of which 35,142 unique barcodes were submitted for further review by an experienced team of nutritionists. Products were excluded from the study due to missing information or nutrient errors on package labeling (*n* = 1339) or if they did not fit any SEARO category assignment (*n* = 1921 no category; *n* = 204 variety packs; *n* = 159 baby food). A total of 31,519 products could be assigned to a category of the SEARO NPM. The allocation of NPM thresholds requires key nutrient information to be present on the label, however the reporting of all nutrients is not mandatory in India. Consequently 19,722 products were missing nutrient information necessary to apply NPMs. All NPM thresholds were based on the nutrient composition of the foods “as consumed”. However many products report nutrients “as sold”, so all non-ready-to-drink beverages were reconstituted before applying the NPMs (i.e., for concentrates and powders, nutrients are diluted with water according to package instructions) and 1296 food products were excluded from the NPM due to lack of detailed preparation instructions necessary for reconstitution. A sample of 10,501 food and beverage products had sufficient “as available to be consumed” nutrient information for comparison with the NPM criteria.

### 2.2. Applying the SEARO and CWO NPM Criteria

The SEARO NPM criteria includes nutrient thresholds for total fat, saturated fat, total sugar, added sugar, sodium, or energy (kcal) based on food category. Specific threshold details are presented in Appendix A Table A1. In addition, any product with added non-nutritive sweetener (NNS) met the criteria for the SEARO NPM. The CWO was implemented in three phases, in 2016, 2018, and 2019, with nutrient thresholds more stringent for each phase [19]. The Phase 3 CWO 2019 thresholds were applied to the current study and include nutrient thresholds for sugar, sodium, saturated fat, and energy for foods and beverages containing added sugar, added sodium, or added saturated fat, but are not based on specific food categories. Details for the CWO are presented in Appendix A Table A2.

### 2.3. Analysis

The main aim of the current study is to ascertain the percentage of packaged foods and beverages that receive at least one warning label for either the SEARO or CWO NPM according to the relevant criteria. Percentages of products meeting the criteria for at least one warning label are presented for the total sample and by food category.

## 3. Results

The SEARO model includes 25 food categories. The number of products, organized by SEARO food category, and the percentage of foods and beverages meeting the SEARO and CWO NPM criteria is presented in Table 1. Seventeen food categories including more than 100 products (ranging from 111 to 1377) with sufficient nutrient information to apply NPMs were identified. This is in contrast to the Nutrition Alchemy data which included only two food categories with more than 100 products, with most categories including 20 products or less for their analysis of the proportion of high in fat, salt, and sugar (HFSS) foods. The current study did not have any products for only one category, 5C fish-based products, due to insufficient information provided on packaging labels.

Overall, 68% of the products met the SEARO criteria for at least one warning label on the front of the package, and 63% of products met the CWO criteria for at least one warning label.

Over 90% of foods met the criteria for at least one SEARO nutrient threshold in six categories: confectionary (99%), fine bakery (100%), processed nuts (95%), frozen dairy desserts (96%), curded dairy desserts (94%), and processed meat (97%). In contrast, less than 50% of foods met the criteria for at least one SEARO nutrient threshold in seven categories: cereals (37%), fats and oils (41%), pasta (33%), fresh/frozen meat (20%), fresh/frozen fruits/vegetables (0%), processed fruits/vegetables (30%), and soybean products (17%).

Among beverages, the majority of juices (76%), dairy drinks (74%), and flavored waters (88%) met the criteria for high in sugar SEARO criteria (i.e., the nutrient cutoff threshold). Other beverage categories were less likely to meet the SEARO criteria for at least one warning label (9% coffee/tea; 39% cereal/nut based beverages).

Table 2 presents the proportion of products by food category meeting the SEARO criteria for number of warning labels (0, 1, 2, 3, or more). Overall, one-third of products would have three or more warning labels, 15% would have two warning labels, 22% would have one warning label, and one-third would have no warning labels under the SEARO NPM. The SEARO NPM results by each nutrient of concern by food category are presented in Supplementary Table S1.

Comparing the NPM results, in general products are less likely to meet the criteria based on the Chilean warning labels compared with the SEARO model. The largest difference is for dairy beverages, where only 35% meet the Chilean Warning Octagon (CWO) criteria compared to 74% for SEARO. One of the major differences between the two NPMs is that CWO requires information on added ingredients for each nutrient of concern before evaluating the nutrient threshold, whilst the SEARO model does not have this same requirement. For example, dairy beverages will be evaluated for the sugar threshold under the CWO only when added sugar is included in the ingredient list, but the sugar thresholds of the SEARO NPM are evaluated among all dairy beverages regardless of ingredients. Thus, the free-sugar values in dairy may exceed the CWO sugar threshold but if there is no added sugar in the product it will not meet criteria for a warning label.

Table 3 presents the results for the Nutrition Alchemy study. In the report we see that only one food category had more than 200 foods and most have fewer than 20 (16 categories). The Nutrition Alchemy data shows 95.5% would have one or more warning labels. Clearly this set of data represents a highly selective set of foods and beverages, as 62.5% have 3 or more high level warning signs. The contrast is significant. The numbers with one, two, and three or more warning labels are: 31.6, 21.8, and 31.6%, respectively. The Nutrition Alchemy report showed for one, two, and three warning labels values of 14.4%, 18.3%, and 62.5%, respectively. In other words, the Nutrition Alchemy dataset allocated warning labels to 62.5% of products compared to about half of that for the sample analyzed by the authors.

## 4. Discussion

This study highlights two key issues: first and foremost, the Indian food supply of packaged and processed foods is rapidly becoming dominated with ultra-processed unhealthy products high in sugar, sodium, and saturated fat. There is a clear need to provide consumers with information through packaging labels to aid in their understanding of the nutrient contents of their food. Second, the recent evaluation of nutrient content based on products provided by the food industry to the Nutrition Alchemy was a small, unrepresentative sample which likely overestimated the percentage of products that would receive a warning label for nutrients of concern. This biased sample with near-universal coverage of products with warning labels does not provide an accurate assessment in the value of NPMs to properly inform consumers.

The Nutrition Alchemy report was released in January 2021 [26]. That study utilized a dataset with a small sample of 1306 products; it is unclear how these products were selected for evaluation. In contrast, the data used in the current study was collected from products available in the Indian market over the last six years consisting of 31,519 products across 25 food categories. Because India does not require sugar to be reported on food labels, only 10,501 products of the sample had adequate nutrition information to evaluate NPMs. Given the large number of products with insufficient information, it is important to note those products that currently provide inadequate information for research purposes, but more importantly to acknowledge that they also do not provide such essential information to consumers. In other countries, nutrient threshold-based regulations typically require all nutrients to be reported on food labels. Products that do not report required nutrients are then subject to the same tax or marketing restrictions as those products who do report and meet criteria to receive warning labels [27]. However, even the subset of data with adequate nutrition information is significantly greater (about eight times larger) than the small 1306 items in the Nutrition Alchemy study. The current study included 17 food categories each with over 100 products, compared with the FSSAI and Nutrition Alchemy study in which the majority of categories had less than 20 products, and only two categories containing over 100 products.

The discrepancy between a sample of 1306 products with 96% exceeding SEARO nutrient thresholds, and a sample of 10,501 products with 68% exceeding SEARO nutrient thresholds highlights the issue of using small industry-selected datasets, as in the Nutrition Alchemy report, as a standard for judging the SEARO NPM. In addition, 62.5% (*n* = 820 products) of the Nutrition Alchemy sample met criteria for three or more warning labels. In contrast, only 31.6% (*n* = 3316) met criteria for three or more warning labels in the current study. These results underscore the fact that the foods provided to the Nutrition Alchemy study by the industry were heavily weighted towards products high in sugar, sodium, and saturated fat, potentially leading to the misinterpretation of the SEARO cutoffs which allowed only 4% of products to have no FOP as too stringent to be useful in policy making.

We do not make this statement lightly. Aside from research conducted on appropriate NPMs for Mexico, Brazil, Colombia, Jamaica, and South Africa, no other sample of food products reported such high proportions of warning labels [28,29,30,31,32]. In all these countries applying the Chilean Phase 3 criteria, the proportion of foods meeting the criteria for warning labels is comparable to the SEARO NPM for India. 

## 5. Conclusions

Overall, using a much larger and more representative sample of food products, this study found that 68% of food products would receive a high-level warning label and 32% would not receive a warning label. This is before the massive reformulation of products that typically occurs after any warning label regulation is implemented [33]. The food industry gave the Nutrition Alchemy the most unhealthy foods possible for selection and thus only 4.5% of these products did not receive a warning label within their study, highlighting yet another way in which the food industry can attempt to manipulate public policy decisions regarding the provision of healthier foods for infants and preschoolers. This study shows that he SEARO NPM criteria and nutrient thresholds are appropriate and important for use by the Indian government’s FSSAI. India has one of the worst food supplies in the world and it must improve if the government is to make an impact in reducing the prevalence of obesity, diabetes, and other nutrition-related noncommunicable diseases [11].

## Figures and Tables

**Table 1:** The percentage of foods and beverages meeting the WHO South-East Asia Region Organization (SEARO) and Chilean Warning Octagon (CWO) nutrient profile modeling (NPM) criteria, by SEARO food category.

SEARO Food Category Code	SEARO Food Category Name	Total N ^1^	N with Nutrient Information ^2^	Meets at Least One Criteria for WHO SEARO NPM ^3^		Meets at Least One Criteria for CWO NPM ^4^	
				N ^5^	%	N ^5^	%
	Total	**31,519**	**10,501**	**7185**	**68.4**	**6623**	**63.0**
1	Confectionary	3148	1377	1368	**99.3**	1333	**96.8**
2	Cakes, sweet biscuits, pastries	2669	667	667	**100**	666	**99.8**
3	Bread and bread products	1050	184	123	**66.8**	109	**59.2**
4	Cereals	2619	1008	377	**37.4**	413	**40.9**
5A	Potato, cereal, or starch-based (from roots, tuber, or legumes), and animal-based (from skin) foods	4092	1312	1312	**100**	1285	**97.9**
5B	Processed nuts	1022	478	454	**94.9**	249	**52.1**
5C	Fish-based foods	4	0	-	**-**	-	**-**
6A	Juices	517	235	180	**76.5**	124	**52.7**
6B	Milk- and dairy-based drinks	905	228	169	**74.1**	80	**35.0**
6C	Water-based flavored drinks	2123	783	691	**88.2**	583	**74.4**
6D	Coffee, coffee substitutes, tea, herbal infusions	1883	1418	132	**9.3**	69	**4.8**
6E	Cereal, grain, tree nut-based beverages	249	155	61	**39.3**	55	**35.4**
7	Frozen dairy-based desserts and edible ices	595	128	123	**96.0**	118	**92.1**
8	Curded dairy-based desserts	426	111	105	**94.5**	102	**91.8**
9	Cheese and analogues	626	200	178	**89**	129	**64.5**
10	Composite foods (prepared foods)	1219	160	114	**71.2**	86	**53.7**
11	Fats and oils, and fat emulsions	1715	204	84	**41.1**	21	**10.2**
12	Pasta, noodles, and similar products	630	9	3	**33.3**	3	**33.3**
13	Fresh and frozen meat, poultry, game, fish, and seafood products	20	20	4	**20**	0	**0**
14A	Processed meat, poultry, and game products	466	61	59	**96.7**	47	**77.0**
14B	Processed fish and seafood products	216	61	43	**70.4**	31	**50.8**
15	Fresh and frozen fruits and vegetables, and legumes	89	14	0	**0**	0	**0**
16	Processed fruits and vegetables	2448	849	255	**30.0**	520	**61.2**
17	Solid-form soybean products	86	6	1	**16.6**	0	**0**
18	Sauces, dips, and dressings	2702	833	682	**81.8**	600	**72.0**

^1^ Total N includes products downloaded from the Mintel Global New Product Database from 2015–2020 in the Indian market that could be assigned to a SEARO food category. ^2^ N with nutrient information includes products with sufficient “as consumed” nutrient information for both the SEARO and CWO NPM criteria. ^3^ Product meets criteria for one or more nutrient thresholds according to the South-East Asia Region Organization (SEARO) nutrient profile model (NPM) for that SEARO category as detailed in Appendix A Table A1. ^4^ Product meets criteria for one or more Chilean Warning Octagon (CWO) Phase 3 NPM nutrient thresholds as detailed in Appendix A Table A2. ^5^ The number of foods with complete nutrient data needed for both the SEARO NPM and the Chilean warning label NPM.

**Table 2:** The percentage of foods and beverages meeting the WHO South-East Asia Region Organization (SEARO) nutrient profile modeling criteria for one or more warning labels, by SEARO food category.

SEARO Food Category Code	SEARO Food Category Name	N with Nutrient Information ^1^	Percent of Products Meet SEARO Criteria			
			No Warning Label	1 Warning Label	2 Warning Labels	3 or More Warning Labels
	Total	**10,501**	**31.6**	**21.8**	**15.04**	**31.6**
1	Confectionary	1377	0.7	1.67	10.31	87.36
2	Cakes, sweet biscuits, pastries	667	0.0	0.30	1.65	98.05
3	Bread and bread products	184	33.2	39.13	26.63	1.09
4	Cereals	1008	62.6	15.97	20.44	0.99
5A	Potato, cereal, or starch-based (from roots, tuber, or legumes), and animal-based (from skin) foods	1312	0.0	0.23	4.80	94.97
5B	Processed nuts	478	5.0	38.28	56.07	0.63
6A	Juices	235	23.4	28.09	48.51	0.00
6B	Milk- and dairy-based drinks	228	25.9	65.79	8.33	0.00
6C	Water-based flavoured drinks	783	11.8	82.63	5.49	0.13
6D	Coffee, coffee substitutes, tea, herbal infusions	1418	90.7	8.96	0.35	0.00
6E	Cereal, grain, tree nut-based beverages	155	60.6	36.77	2.58	0.00
7	Frozen dairy-based desserts and edible ices	128	3.9	17.97	31.25	46.88
8	Curded dairy-based desserts	111	5.4	26.13	46.85	21.62
9	Cheese and analogues	200	11.0	16.50	46.00	26.50
10	Composite foods (prepared foods)	160	28.8	39.38	15.63	16.25
11	Fats and oils, and fat emulsions	204	58.8	32.84	8.33	0.00
12	Pasta, noodles, and similar products	9	66.7	33.33	0.00	0.00
13	Fresh and frozen meat, poultry, game, fish, and seafood products	20	80.0	20.00	0.00	0.00
14A	Processed meat, poultry, and game products	61	3.3	32.79	63.93	0.00
14B	Processed fish and seafood products	61	29.5	27.87	31.15	11.48
15	Fresh and frozen fruits and vegetables, and legumes	14	100.0	0.00	0.00	0.00
16	Processed fruits and vegetables	849	70.0	29.68	0.35	0.00
17	Solid-form soybean products	6	83.3	16.67	0.00	0.00
18	Sauces, dips, and dressings	833	18.1	34.45	44.18	3.24

^1^ N with nutrient information includes products with sufficient “as consumed” nutrient information for both the SEARO and Chilean Warning Octagon nutrient profile model criteria.

**Table 3:** Number (%) of products (per category) containing 0 nutrients/1 nutrient/2 nutrients/3+ nutrients high in sugar, saturated fat, or sodium (HFSS) foods and beverages.

Food Safety and Standards Agency of India Sub-Category Code (Level 3/4)	Number of HFSS Nutrients							
	0 (Non-HFSS)		1		2		3±	
	*n*	%	*n*	%	*n*	%	*n*	%
Totals	**59**	**4.5%**	**188**	**14.4**	**239**	**18.3**	**820**	**62.5**
1.1.2. Dairy-based drinks—flavoured milk and/or fermented	7	9.3	47	62.7	19	25.3	2	2.7
1.6.4 Cheese and Analogues	0	0.0	0	0.0	0	0.0	5	100.0
1.7 Dairy-based desserts	0	0.0	3	2.1	10	6.8	133	91.1
2.2.2 Fat emulsions, mainly water-in-oil	0	0.0	4	100.0	0	0.0	0	0.0
2.4.1 Cocoa-based spreads, including fillings	0	0.0	5	100.0	0	0.0	0	0.0
3. Edible Ices	0	0.0	0	0.0	16	100.0	0	0.0
4.1.2.5 & 4.1.2.9 Jams, Jellies, and Marmalades, and fruit-based desserts, fruit Cheese, including fruit-flavoured, water-baseddesserts	0	0.0	8	100.0	0	0.0	0	0.0
4.2.2.1 Frozen vegetables	4	26.7	11	73.3	0	0.0	0	0.0
4.2.2.5 Vegetables (seed purees and spreads)	6	60.0	0	0.0	4	40.0	0	0.0
4.2.2.6 Vegetables (seed pulps and preparations)	3	42.9	3	42.9	0	0.0	1	14.3
5.1.3 and 5.1.4 Cocoa and chocolate products	0	0.0	0	0.0	0	0.0	94	100.0
5.2.1 Hard candy	0	0.0	5	9.1	0	0.0	50	90.9
5.2.2 and 5.2.3 Soft candy, nougats, and marzipans	0	0.0	0	0.0	0	0.0	46	100.0
5.3 Chewing gum	5	26.3	0	0.0	0	0.0	14	73.7
5.4 Decorations (e.g., for fine bakery wares), toppings (non-fruits), and sweet sauces	0	0.0	0	0.0	1	25.0	3	75.0
6.3 Ready-to-eat cereals, breakfast cereals, including rolled oats (sweet)	1	1.7	18	30.5	10	16.9	30	50.8
6.3 Ready-to-eat cereals, breakfast cereals, including rolled oats (salty)	2	12.5	10	62.5	3	18.8	1	6.3
6.4.3 Pasta, noodles, and similar products (e.g., rice paper, rice vermicelli, soybean pasta, and noodles)	0	0.0	0	0.0	3	8.1	34	91.9
6.7 Pre-cooked or processed cereals/grains/legume products	0	0.0	12	41.4	14	48.3	3	10.3
6.8.1 Soybean-based beverages	1	10.0	9	90.0	0	0.0	0	0.0
7.1.2 & 7.1.4 Bread and ordinary bakery wares and mixes	0	0.0	0	0.0	0	0.0	22	100.0
7.2.1 Fine bakery wares (sweet, salty, savory) and mixes (biscuits, cookies)	0	0.0	0	0.0	0	0.0	73	100.0
7.2.1 Fine bakery wares (sweet, salty, savory) and mixes (cream sandwich biscuits)	0	0.0	0	0.0	0	0.0	55	100.0
7.2.1 Fine bakery wares (sweet, salty, savory) and mixes (cakes)	0	0.0	0	0.0	0	0.0	20	100.0
7.2.2 Other fine bakery products	0	0.0	0	0.0	0	0.0	21	100.0
7.2.3 Mixes for fine bakery wares	0	0.0	0	0.0	0	0.0	5	100.0
12.5.2 Mixes for soups and broths	14	82.4	3	17.6	0	0.0	0	0.0
12.6.2 & 12.6.3 Non-emulsified sauces and mixes for sauces and gravies	3	21.4	5	35.7	1	7.1	5	35.7
14.1.2 Fruit and vegetable juices	2	9.5	17	81.0	2	9.5	0	0.0
14.1.4.1 Carbonated water-based flavoured drinks	8	28.6	0	0.0	20	71.4	0	0.0
14.1.4.2 Non-carbonated water-based flavoured drinks	1	0.8	6	5.1	109	92.4	2	1.7
14.1.4.3 Concentrates (liquid or solid) for water-based flavoured drinks	0	0.0	2	9.1	20	90.9	0	0.0
14.1.5 Coffee, coffee substitutes, tea, herbal infusions	2	12.5	14	87.5	0	0.0	0	0.0
15.1 Snacks and savories—potato, cereal, flour, or starch-based (from roots and tubers, pulses and legumes) foods	0	0.0	1	0.6	1	0.6	177	98.9
15.2 Processed nuts, including coated nuts and nut mixtures	0	0.0	4	44.4	5	55.6	0	0.0
16 Prepared Foods	0	0.0	1	3.8	1	3.8	24	92.3

% = *n*/Number of products in each sub-category × 100 Source: the Nutrition Alchemy.

## Data Availability

The data will be available for use but not possession by request from BPBH and DM. Due to legal constraints established by Mintel with UNC, the data will be put on a UNC server for any user to access and use.

## Supplementary Materials

The following are available online at https://www.mdpi.com/article/10.3390/nu13082799/s1, Table S1: Number and proportion of packaged food and beverage products meeting the criteria for regulation under the SEARO nutrient profile model, by SEARO category (of those with sufficient nutrient info).

## Appendix A

**Table A1:** WHO South-East Asia Region Organization (SEARO) Nutrient Profile Model (NPM) nutrient threshold criteria by SEARO food category.

SEARO Food Category Code	SEARO Food Category Name	Examples of Food Items	Nutrient Thresholds					
			Total Fat (g)	Saturated Fat (g)	Total Sugars (g)	Added Sugars (g)	Sodium (g)	Energy (kcal)
1	Confectionery	Cocoa/chocolate bars and spreads, including imitations and chocolate substitutes; hard, soft, and chewy candies; chewing gum, Indian sweets, sweet sauces, topping sauces, and creamy, sweet, and traditional desserts	8.0	N/A	6.0	N/A	N/A	230
2	Cakes, sweet biscuits, pastries	Cakes, cookies, pies, doughnuts, sweet rolls, muffins, macaroons, biscuits, pancakes (ready-to-eat form)	8.0	N/A	6.0	N/A	0.25	230
3	Bread and bread products	Bread and rolls, pita, naan, rotis, steamed bread, steamed buns, crackers, mixes for making bread, and ordinary bakery wares	8.0	N/A	6.0	N/A	0.25	N/A
4	Cereals	Whole, broken, or flaked grains of rice and other cereals (dalia-broken wheat); rice-based, wheat-based, or maize-based breakfast cereals of all flavours; oat meal, mueslis, granola, and muesli bars; cereal bars, rice cakes	12.0	N/A	9.0	N/A	0.35	N/A
5A	Potato, cereal, or starch-based (from roots, legumes, or tubers), and animal-based (from skin) foods	Popcorn and maize corn, savory biscuits, crackers, other snacks made from rice, maize, wheat, dough, potato, cassava (i.e., chips, crisps),varieties of namkeen, papadums	8.0	N/A	N/A	0	0.05	230
5B	Processed nuts	Nuts and mixed nuts (including with fruit content)	N/A	N/A	N/A	0	0.05	N/A
5C	Fish-based foods	fish-based snacks (savory crackers with fish, fish products, or fish flavoring NOT fish jerky)	N/A	N/A	6.0	N/A	0.25	230
6A	Juices	100% fruit and vegetable juices prepared from direct extraction or reconstituted from concentrate	N/A	N/A	6.0	0	N/A	N/A
6B	Milk- and dairy-based drinks	Milk, butter milk, flavoured dairy-based milk, fermented dairy-based milk, e.g., chocolate milk, strawberry milk, cocoa, eggnog, drinking yoghurt, whey-based drinks. Milk means milk from animals such as cow, buffalo, goat, etc.	7.0	N/A	N/A	0.0	N/A	N/A
6C	Water-based flavoured drinks	Sport, energy, electrolyte drinks, carbonated, and non-carbonated water-based flavoured drinks, jaljeera, concentrates (liquid or solid) in or calculated as ready-to-drink form	N/A	N/A	2.0	N/A	0.30	N/A
6D	Coffee, coffee substitutes, tea, herbal infusions	Coffee, coffee substitutes, tea, herbal infusion in or calculated as ready-to-drink form	N/A	N/A	2.0	N/A	N/A	N/A
6E	Cereal, grain, tree nut-based beverages	Cereal, grain, and tree nut-based beverages produced from the extracts of cereals, beans, pulses, and tree nuts, e.g., rice-, almond-, soybean-, and oat-based beverages.	N/A	N/A	6.0	N/A	0.20	N/A
7	Frozen dairybased dessertsand edible ices	Ice cream, ice milk, frozen flavouredyoghurt, iced lollipops, and sorbets	8.0	N/A	12.0	N/A	0.10	230
8	Curded dairy-based desserts	Dairy-based products that have been curded by fermentation, acid, enzyme, heat, etc., and flavoured with sugar and other ingredients. Examples are flavoured cream-type yoghurt, jellied milk, caramel pudding, butterscotch pudding, chocolate mousse, khoa, peda, burfee, and gulab jamun.	7.0	N/A	6.0	N/A	0.10	230
9	Cheese and analogues	Un-ripened or ripened cheese, whey cheese, processed cheese, cheese analogues, whey protein cheese that can be classified based on physical characteristics as hard (e.g., parmesan), semi-hard (e.g., cheddar), medium-hard (e.g., edam), semi-soft and soft (e.g., mozzarella, paneer, cottage), as well as serving style such as sliced, grated, or spreadable	20.0	N/A	N/A	0.0	0.60	N/A
10	Composite foods (preparedfoods)	Mixtures of multiple components (e.g., meat, sauce, grain, cheese, vegetables). These include foods that require minimal preparation heating, thawing, rehydrating), or ready-to-serve meals from restaurants. Examples: frozen and chilled ready meals, hamburgers, fried chicken, pizzas, lasagna, ready-made sandwiches, soups, instant noodles, instant porridge, steamed pork buns, dumplings, burgers in buns, ready meals, soups	8.0	3.5	9.0	N/A	0.35	N/A
11	Fats and oils, and fat emulsions	Butter oil, anhydrous milk fat, ghee, vegetable oils and fats, lard, tallow, fish oils and other animal fats, butter, margarine, and similar products. Examples: cooking oils from plant and animal sources, butter, margarine, fat blends. Fat spreads	N/A	35.0	N/A	N/A	0.10	N/A
12	Pasta, noodles, and similar products	Fresh, precooked, or dried noodles, pasta, and similar products; rice paper, rice noodles, vermicelli made from wheat, tapioca, sago, legume etc. (cooked as ready to eat)	3.0	N/A	N/A	N/A	0.25	N/A
13	Fresh and frozen meat, poultry, game, fish, and seafood products	Fresh and frozen meat, poultry, game, mollusks, crustaceans, echinoderms in the forms of wholepieces, cuts/fillet, comminuted/minced/creamed. Examples: beef, pork, chicken, lamb, goat, tuna, mackerel, catfish, shrimp etc.	15.0	N/A	N/A	N/A	N/A	N/A
14A	Processed meat, poultryand game products	Non-heat and heat-treated whole pieces or cuts or commuted meat poultry and game that have been cured/cured and dried, or fermented. Examples include smoked ham, salted dried meat, salami, sausage, bacon, corned beef, smoked duck, canned meats, chicken nuggets, beef or chicken patty, pork rind.	8.0	N/A	N/A	N/A	0.40	N/A
14B	Processed fish andseafood products	Frozen battered, cooked and/or fried, smoked, dried, fermented, and/or salted, semi-preserved by pickling or brining, fully-preserved by canning or fermentation of fish and sea foods.Examples: salted fish and seafood, brined fish, salted fish in oil, fermented fish and seafood, anchovies, shrimp paste, canned tuna, sardine, or mackerel, smoked fishes, dried shrimp, fish balls, fish fingers	8.0	3.0	N/A	N/A	0.40	N/A
15	Fresh or frozen fruits and vegetables; legumes	Fruits, vegetables, mushrooms and fungi, roots and tubers, pulses and legumes, nuts and seeds, seaweed	N/A	N/A	N/A	N/A	N/A	N/A
16	Processed fruits and vegetables	Dried, canned, or bottled jam, jellies, marmalades; packed in vinegar, oil or brine, pickles, spreads, candied, pulp, purees, topping, milk, fermented, fillings, cooked forms of fruits and vegetables. Examples: fruits and vegetables in vinegar, oil, or brine; dried fruits; marmalade or jams; canned fruits, vegetables, and legumes; dried mushrooms; preserved or pickled fruits and vegetables; pickled tea leaves, peanut butter	N/A	N/A	N/A	N/A	0.40	N/A
17	Solid-form soybean products	Soybean-based beverages, soybean curd (tofu), semi-dehydrated tofu, dehydrated tofu (kori tofu), fermented soybeans (natto, tempeh), other soybean protein products (soya nuggets and textured vegetable protein)	12.0	N/A	5.0	N/A	0.10	N/A
18	Sauces, dips, and dressings	Emulsified and non-emulsified mixes as concentrated, clear sauces and similar products, soybean-based seasoning, and condiments. Examples: mayonnaise, salad dressing, onion dips, tomato ketchup, colored ketchup, gravy, cheese sauce, cream sauce, bouillon cubes, seasoning powder, fermented and nonfermented soy sauces, fish sauce, sweet chili sauce, spaghetti sauce, BBQ sauces, chili paste, chutney, and marmite	12.0	N/A	10.0	N/A	0.30	N/A

**Table A2:** Chilean Warning Octagon (CWO) Nutrient Profile Model (NPM) nutrient threshold criteria for foods and beverages based on Phase 3 (2019) criteria.

Nutrient per 100 g or mL ^1^	Liquids ^2^ (mL)	Solids ^3^ (Grams)
Calories (kcal)	70	275
Sodium (mg)	100	400
Total sugar (g)	5	10
Saturated fat (g)	3	4

^1^ Nutrient threshold criteria applies to all package foods and beverages with added sugar, added sodium, or added saturated fat. ^2^ Liquids includes any product that presents their nutritional composition “as consumed” per 100 mL. ^3^ Solids includes any product that indicates their nutritional composition “as consumed” per 100 g.
